# An Uncommon Cause of Syncope and Left Atrial Appendage Thrombus: Speech-Induced Atrial Tachycardia

**DOI:** 10.1016/j.jaccas.2024.102470

**Published:** 2024-08-21

**Authors:** Shiro Miura, Wataru Nagahori, Hirofumi Mitsuyama, Takehiro Yamashita

**Affiliations:** Department of Cardiology, Sapporo Kojinkai Memorial Hospital, Sapporo, Japan

**Keywords:** atrial arrhythmia, computed tomography, left atrial thrombosis, syncope

## Abstract

An 83-year-old, previously healthy woman experienced frequent episodes of syncope following conversations. Speech-induced atrial tachycardia complicated by left atrial appendage thrombus was diagnosed as a potential etiology. She was successfully treated via catheter ablation. This is the first case report suggesting an association between arrhythmia, syncope, and atrial thrombus formation.

## Graphical Abstract

**Figure:**
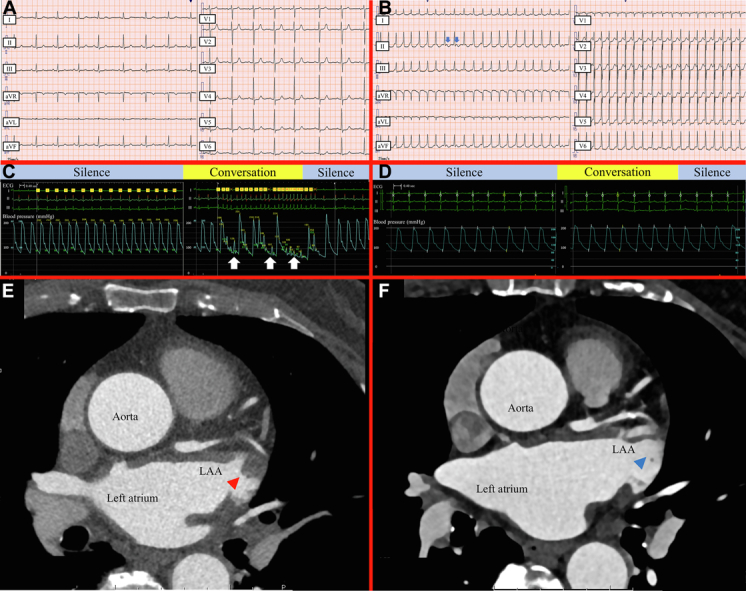

An 83-year-old woman was referred for frequent episodes of syncope following conversations over the past 3 months. Syncope occurred during standing and sitting, but not while lying down. Her past medical history was unremarkable except for well-controlled hypertension. Vital signs revealed a heart rate of 81 beats/min, a blood pressure of 142/76 mm Hg, a respiratory rate of 14 breaths/min, a body temperature of 36.5°C, and an oxygen saturation of 98%. The physical examination was unremarkable. The 12-lead electrocardiogram (Figure 1A) and echocardiogram on admission were normal. A brain magnetic resonance imaging revealed no evidence indicating a transitory ischemic attack, silent stroke, or a possible genesis of syncope. According to 24-hour Holter monitoring, transient supraventricular tachycardia (≥3 beats) was frequently recorded in 14% of the total recording time, with the longest episode lasting 25 seconds. With a 14-day cardiac event recorder, atrial fibrillation (AF) was not detected. In-hospital cardiac monitoring revealed that atrial tachycardia (AT) (Figure 1B) triggered by premature atrial complexes could be reproducibly induced when she started speaking, immediately terminating when she stopped speaking. Deep breaths, deglutition, or coughing could not provoke the AT. A simultaneous continuous 12-lead electrocardiogram and invasive blood pressure monitoring in our catheterization laboratory (Figure 1C, Video 1) revealed that the rapid AT was reproducibly induced by her speech and caused a substantial drop in blood pressure along with dizziness. These findings led to the diagnosis of speech-induced AT.^1^ The patient was initially treated with 2.5 mg of bisoprolol daily for 4 weeks, which failed to alleviate her symptoms fully. Therefore, an electrophysiological study with subsequent catheter ablation was scheduled. Contrast cardiac computed tomography (Figure 1E) identified a left atrial appendage thrombus, which was completely resolved after daily administration of edoxaban 30 mg for 3 months (Figure 1F). Cardiac magnetic resonance imaging and Technetium-99m pyrophosphate single photon emission computed tomography showed no remarkable findings suggesting underling cardiomyopathies such as cardiac amyloidosis. An electrophysiological study using the EnSite X EP System (Abbott) (Supplemental Figure 1A) revealed that the earliest focal activation site of the AT was on the septal side of the right atrium, which was successfully ablated (Supplemental Figure 1B). Before discharge, repeated simultaneous continuous 12-lead electrocardiogram and blood pressure monitoring confirmed the absence of speech-induced arrhythmia (Figure 1D, Video 2). At the 3-month visit, she was asymptomatic with no recurrence of AT and no other atrial arrhythmia recorded.

**Figure 1:**
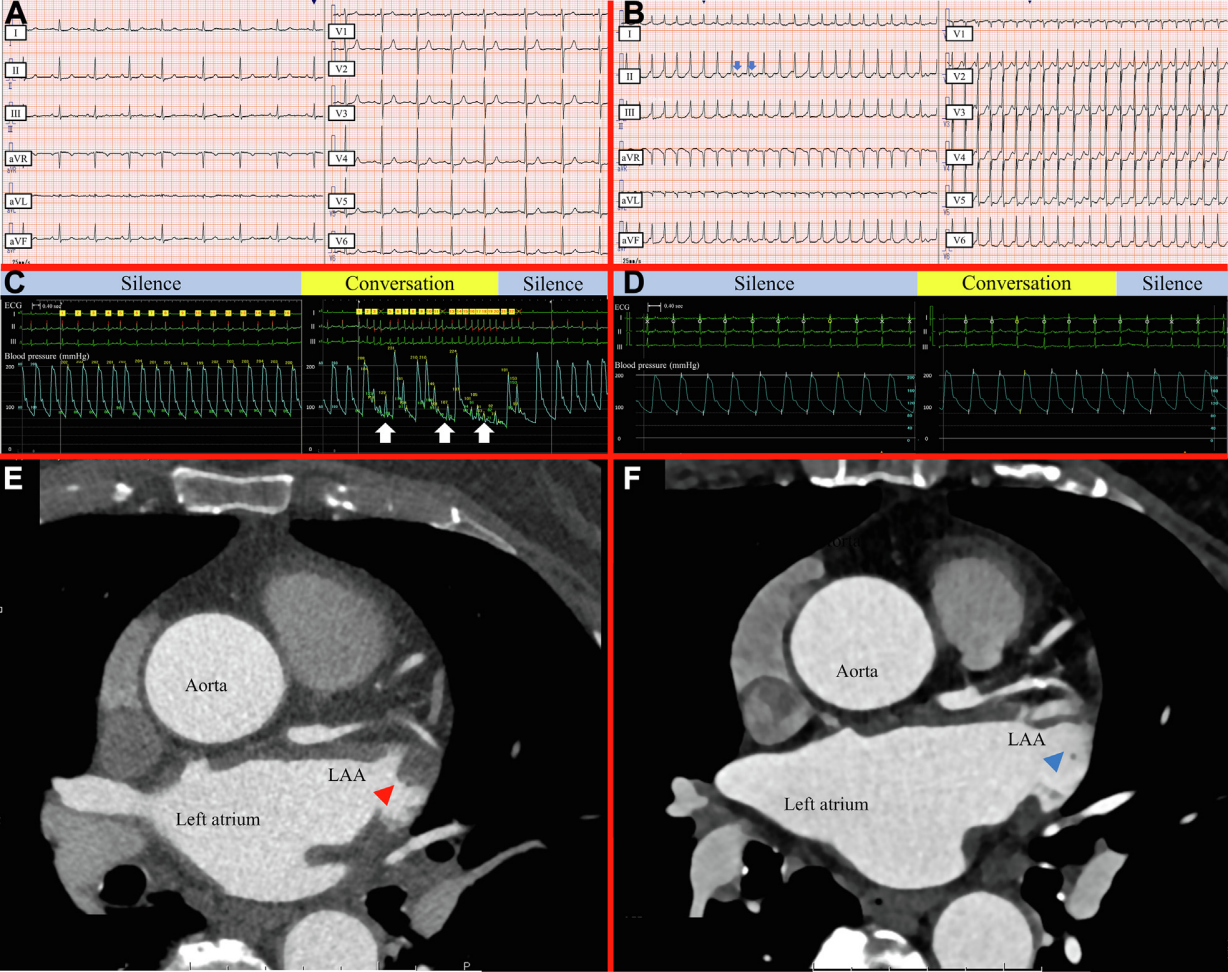
Multimodality Imaging Findings Twelve-lead electrocardiograms show normal sinus rhythm at initial presentation (A) and sustained atrial tachycardia (AT) during conversation (B) with 1:1 conduction at 173 beats/min. Blue arrows indicate positive P waves. Simultaneous continuous 12-lead electrocardiogram and invasive blood pressure monitoring at initial presentation (C) demonstrate an acute drop in blood pressure triggered (white arrows) by rapid AT during conversation from 208/89 mm Hg (heart rate, 83 beats/min) to 73/61 mm Hg (171 beats/min) in the supine position. By contrast, simultaneous monitoring after catheter ablation (D) shows no change in blood pressure and heart rhythm during conversation. Contrast cardiac computed tomography at initial presentation (E) shows a thrombus in the left atrial appendage (LAA) (red arrowhead), whereas one after oral anticoagulant therapy (F) shows complete thrombus resolution (blue arrowhead).

## Discussion

Speech-induced AT is an uncommon, debilitating arrhythmia that can cause palpitations and presyncope during conversations.^1^ Reliable correlation between each episode of speech and subsequent onset of a rapid, narrow complex tachycardia is highly diagnostic. The unique phenomenon can be functionally related to the autonomic nervous system rather than anatomical abnormalities, although the underlying mechanisms remain poorly elucidated.^2^ We believe her syncope, an extremely rare complication of the entity, was most likely linked to vasomotor factors by autonomically mediated focal AT.^3^ We could confirm relationships between symptoms, blood pressure, and AT using a novel approach, that is, simultaneous rhythm and blood pressure monitoring. Pharmacologic therapy, predominantly using beta-blockers, is a safe and accessible option for the initial management of symptoms. Catheter ablation is a widely used, highly effective, and preferred long-term therapy for focal AT when pharmacologic therapy fails.^1^^,^^2^ We identified the septal side of the right atrium, close to anterior right ganglionated plexuses,^2^ as the arrhythmogenic origin and successfully ablated it without reported recurrence. Based on the Holter monitoring analysis, we speculated that the high arrhythmia burden had impaired left atrial appendage function, thereby causing thrombus formation. Nevertheless, the possibility of an undetected AF cannot be ignored, although AF was not recorded after catheter ablation.

## Funding Support and Author Disclosures

The authors have reported that they have no relationships relevant to the contents of this paper to disclose.

## Appendix

Video 1 An initial simultaneous continuous 12-lead electrocardiogram and invasive blood pressure monitoring in the supine position at the catheter laboratory. The monitoring shows premature atrial complexes could be induced by speech initiation, followed by a substantial drop in blood pressure. The arrhythmia terminated and blood pressure recovered immediately after cessation of the conversation.

Video 2 A simultaneous continuous 12-lead electrocardiogram and invasive blood pressure monitoring post-catheter ablation shows no change in blood pressure and heart rhythm.
