# Supplementary material for: An Uncommon Cause of Syncope and Left Atrial Appendage Thrombus: Speech-Induced Atrial Tachycardia
Source: JACC Case Rep. 2024 Aug 21;29(16):102470. doi: 10.1016/j.jaccas.2024.102470 (PMC11405956; doi:10.1016/j.jaccas.2024.102470)

**Supplemental figure 1**

(A) Three-dimensional electroanatomical mapping illustrates that the earliest atrial tachycardia focal activation site (white arrow) is on the septal side of the right atrium. (B) Cine capture image shows mapping and ablation catheter positions.


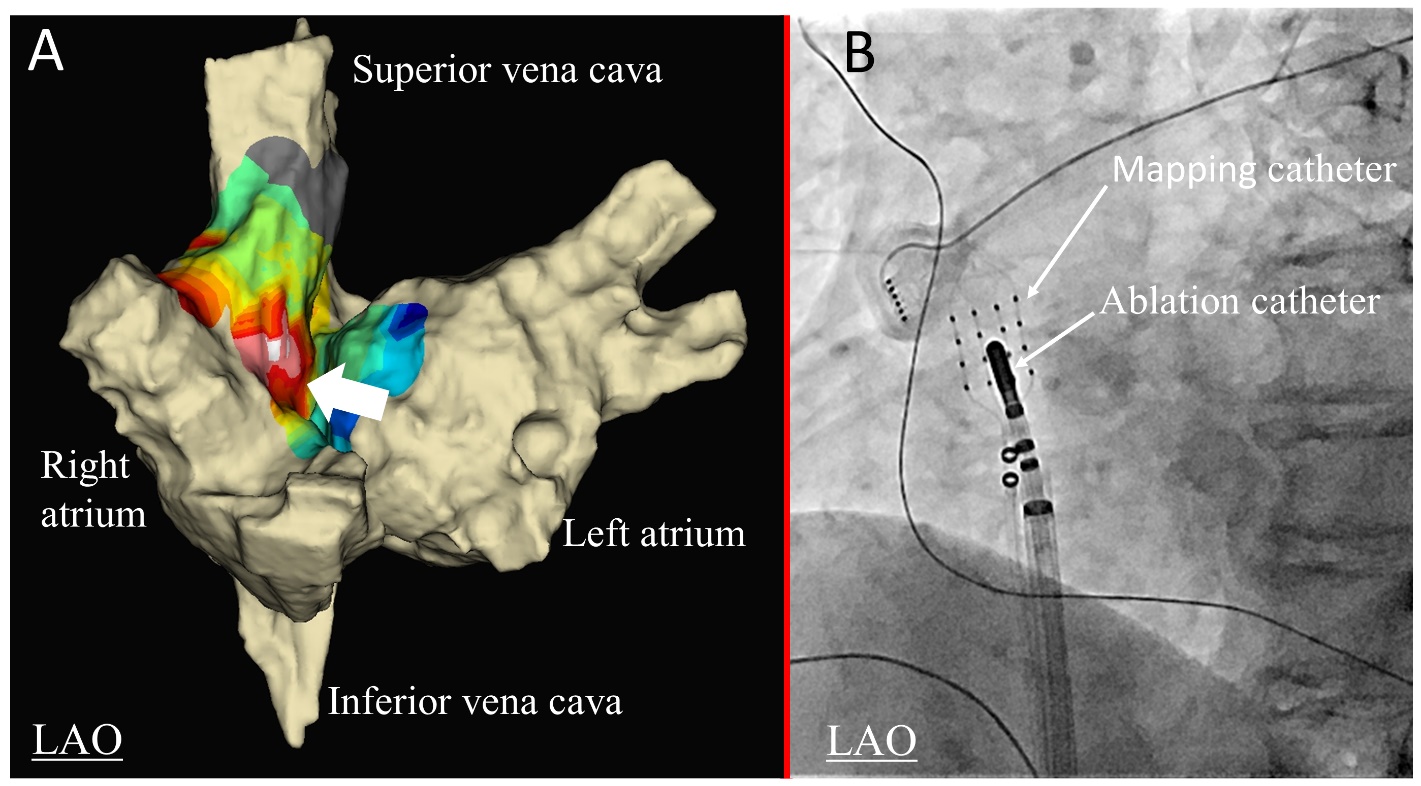

Supplement: Supplemental Figure 1 [file mmc3.docx]
